# Implementation of California COVIDNet – a multi-sector collaboration for statewide SARS-CoV-2 genomic surveillance

**DOI:** 10.3389/fpubh.2023.1249614

**Published:** 2023-10-23

**Authors:** Debra A. Wadford, Nikki Baumrind, Elizabeth F. Baylis, John M. Bell, Ellen L. Bouchard, Megan Crumpler, Eric M. Foote, Sabrina Gilliam, Carol A. Glaser, Jill K. Hacker, Katya Ledin, Sharon L. Messenger, Christina Morales, Emily A. Smith, Joel R. Sevinsky, Russell B. Corbett-Detig, Joseph DeRisi, Kathleen Jacobson

**Affiliations:** ^1^California Department of Public Health, Richmond, CA, United States; ^2^Orange County Public Health Laboratory, Santa Ana, CA, United States; ^3^Theiagen Genomics, Highlands Ranch, CO, United States; ^4^Pathogen Genomics Center, University of California, Santa Cruz, Santa Cruz, CA, United States; ^5^University of California, San Francisco, San Francisco, CA, United States; ^6^Chan Zuckerberg Biohub, San Francisco, CA, United States

**Keywords:** SARS-CoV-2, genomic surveillance, COVID-19, whole genome sequencing, cloud-based computing, data management

## Abstract

**Introduction:**

The SARS-CoV-2 pandemic represented a formidable scientific and technological challenge to public health due to its rapid spread and evolution. To meet these challenges and to characterize the virus over time, the State of California established the California SARS-CoV-2 Whole Genome Sequencing (WGS) Initiative, or “California COVIDNet”. This initiative constituted an unprecedented multi-sector collaborative effort to achieve large-scale genomic surveillance of SARS-CoV-2 across California to monitor the spread of variants within the state, to detect new and emerging variants, and to characterize outbreaks in congregate, workplace, and other settings.

**Methods:**

California COVIDNet consists of 50 laboratory partners that include public health laboratories, private clinical diagnostic laboratories, and academic sequencing facilities as well as expert advisors, scientists, consultants, and contractors. Data management, sample sourcing and processing, and computational infrastructure were major challenges that had to be resolved in the midst of the pandemic chaos in order to conduct SARS-CoV-2 genomic surveillance. Data management, storage, and analytics needs were addressed with both conventional database applications and newer cloud-based data solutions, which also fulfilled computational requirements.

**Results:**

Representative and randomly selected samples were sourced from state-sponsored community testing sites. Since March of 2021, California COVIDNet partners have contributed more than 450,000 SARS-CoV-2 genomes sequenced from remnant samples from both molecular and antigen tests. Combined with genomes from CDC-contracted WGS labs, there are currently nearly 800,000 genomes from all 61 local health jurisdictions (LHJs) in California in the COVIDNet sequence database. More than 5% of all reported positive tests in the state have been sequenced, with similar rates of sequencing across 5 major geographic regions in the state.

**Discussion:**

Implementation of California COVIDNet revealed challenges and limitations in the public health system. These were overcome by engaging in novel partnerships that established a successful genomic surveillance program which provided valuable data to inform the COVID-19 public health response in California. Significantly, California COVIDNet has provided a foundational data framework and computational infrastructure needed to respond to future public health crises.

## 1. Introduction

In early 2020, as SARS-CoV-2 began to spread rapidly around the world, it became clear that an unprecedented, multi-faceted, and coordinated response would be required for this public health crisis. In April 2020, the Governor of California established the California Testing Task Force (CA-TTF)^1^ (1) to address the daunting need to provide COVID-19 testing for the state’s population of nearly 40 million. Akin to actions taken by the United Kingdom (2), the CA-TTF implemented the California SARS-CoV-2 Whole Genome Sequencing (WGS) Initiative, a genomic surveillance program created to track evolution of the virus over time, monitor variant and lineage transmission throughout the state, and characterize outbreaks and clusters of this virus. Objectives of this program included developing a network of public health laboratories (PHLs) with long-term sequencing capabilities, building genomic epidemiology capability at the state and LHJs for real-time public health action, and establishing and maintaining long-term partnerships among LHJs, academic institutions, and other public, non-profit, and private institutions. This endeavor, named “California COVIDNet,” is led by the California Department of Public Health (CDPH) with guidance, support, and input from the Chan Zuckerberg Biohub (CZB) and comprises an exceptional, collaborative network of public, private, and academic laboratories that partnered to scale WGS in response to the COVID-19 pandemic. Herein, we describe the implementation of California COVIDNet, hereafter designated as COVIDNet.

## 2. Materials and methods

### 2.1. Implementation of COVIDNet

**Figure 1:**
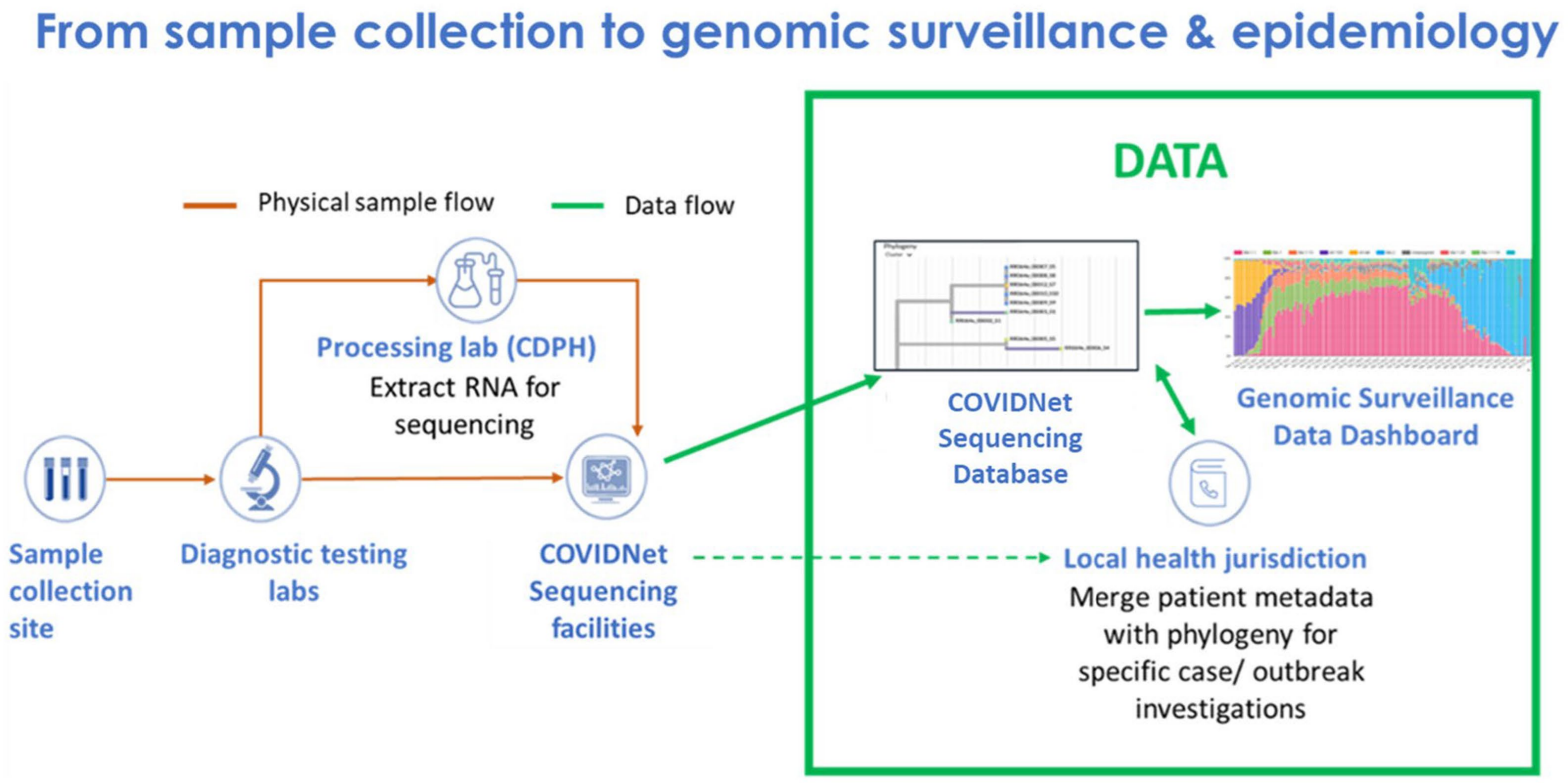
Flow of samples and data from sample collection to genomic sequencing and epidemiology for SARS-CoV-2 genomic surveillance by COVIDNet.

Implementing COVIDNet required establishing systems and processes, many of which did not exist prior to the pandemic, to manage sample data and sample flow (Figure 1). This included (1) data management systems to anonymize and store SARS-CoV-2 positive sample data to ensure patient privacy, (2) cloud-based storage capacity for WGS data, (3) bioinformatics capabilities for sequence analysis, (4) a network of testing sites and laboratories to source random, representative SARS-CoV-2 positive samples throughout the state, and (5) a network of laboratories to process and sequence samples. Also required were the infrastructure, processes, and procedures to receive sequence data from partner laboratories for centralized and standardized bioinformatics processing, quality control, and analyses for transmission of high-quality lineage results to the state’s COVID-19 reporting system and uploading of the data to public repositories. Consultants and contractors with demonstrated expertise in viral evolution, bioinformatics, genomics, and privacy were engaged to advise and guide the execution of many of these processes, including (1) an expert advisory group (see below), (2) a CDPH legal and privacy advisory team, (3) Theiagen Genomics (Highlands Ranch, CO United States), to provide bioinformatics support, including pipelines for data quality control and analysis, lineage reporting and uploading, and cloud-based data storage, and (4) the University of California Santa Cruz (UCSC) Pathogen Genomics Center to develop and implement a system of tools for genomic analyses, including applications for health departments to characterize clusters and outbreaks in their jurisdictions.

### 2.2. Expert panel advisory group

In June 2020, CDPH convened an advisory panel of nationally and internationally recognized experts comprised of 18 distinguished researchers and subject matter experts in genomic sciences, viral evolution, and mathematical modeling. This expert panel was assembled to support, advise, and guide the initial phases of COVIDNet implementation as well as to engage with LHJs in analyzing and interpreting WGS results.

### 2.3. Sequencing and data storage capacity

In the first year of the pandemic, most public health-oriented sequencing of California SARS-CoV-2 positive samples was performed by the CZB in association with California local PHLs, as well as by the SEARCH Alliance in San Diego. Although COVIDNet was conceived in April 2020, scaled-up laboratory operations of COVIDNet did not begin until March 2021. This delay was due to factors related to establishing protocols and practices for specimen and data acquisition, flow, and management for a large and populous state. A considerable amount of time and effort was required to recruit and onboard partner laboratories to source or sequence specimens in the midst of the pandemic. CDPH determined and applied best practices to maintain compliance related to data security and privacy, as well as navigate the challenges of logistics and contracting to implement the computational infrastructure required for bioinformatics analytics. CDPH contracted with Theiagen Genomics, which provided a cloud-based solution to house and analyze viral sequence data via Terra.bio on the Google Cloud Platform (GCP).

### 2.4. Sampling representativeness

**Figure 2:**
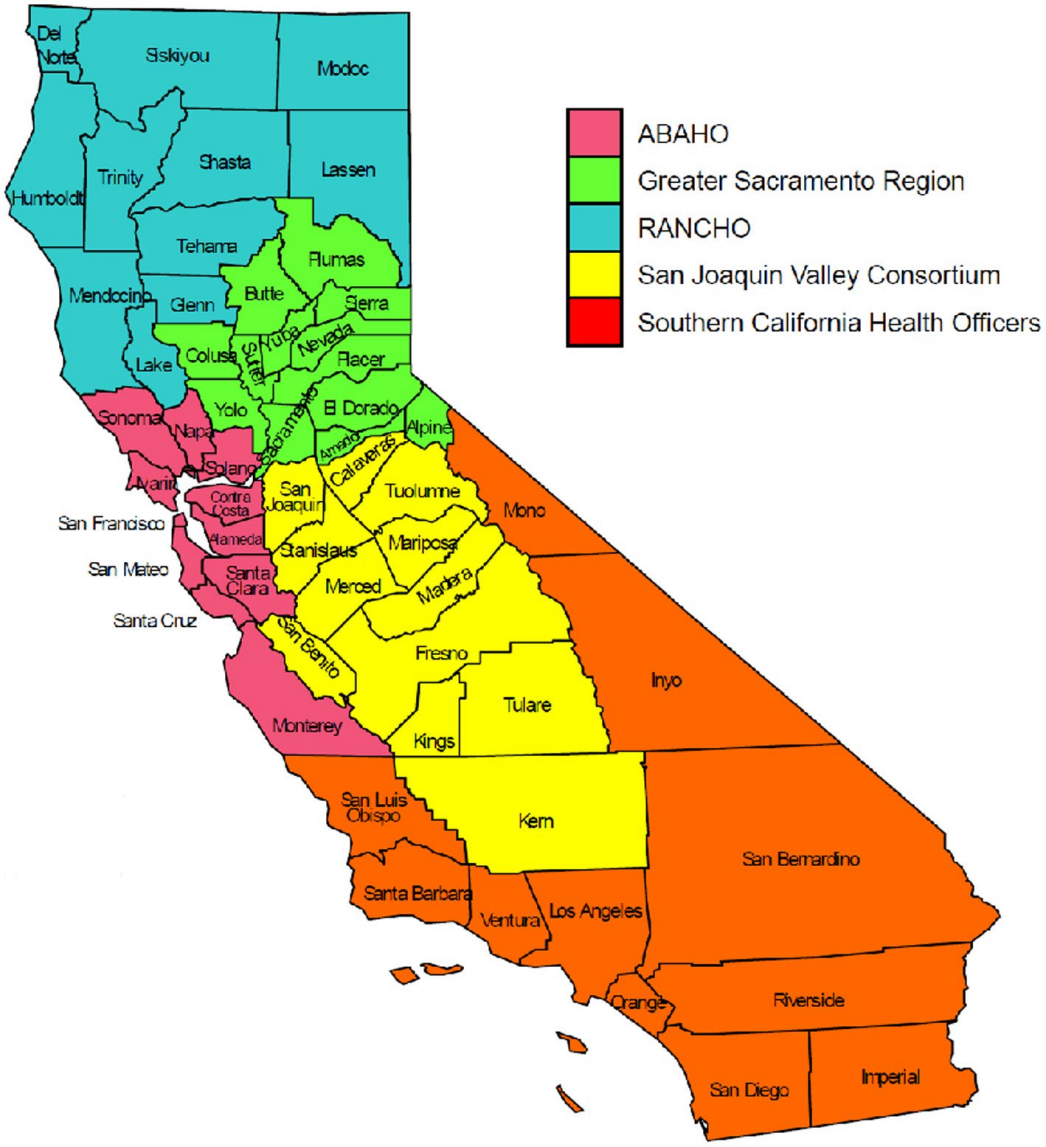
The five California Health Officers Regions. Red: Association of Bay Area Health Officials (ABAHO); Green: Greater Sacramento Region of Health Officers (GSRHO); Blue: Rural Association of Northern California Health Officers (RANCHO); Yellow: San Joaquin Valley Consortium of Health Officers (SJVCHO); Orange: Southern California Health Officers (SCHO). https://www.cdph.ca.gov/Programs/CID/DCDC/Pages/COVID-19/Order-of-the-State-Public-Health-Officer-Hospital-Health-Care-System-Surge-FAQ.aspx (3).

One of the goals of genomic surveillance is to ensure sufficient representativeness of the tested population. Successful genomic surveillance of COVID-19 in California required a sequencing strategy representative of the state’s geography and diverse communities that would yield an accurate estimate of SARS-CoV-2 lineages circulating within the state. Achieving the volume and distribution of sequencing that matched true infection rates in each community was not realistic or feasible due to resource and logistical constraints. To coordinate the public health response related to COVID-19 policies (such as stay-at-home orders, tracking hospitalization and ICU capacity, etc.) across the 61 California LHJs, five Health Officers regions were organized as follows: Rural Association of Northern California Health Officers (RANCHO), Association of Bay Area Health Officials (ABAHO), Greater Sacramento Region of Health Officers (GSRHO), San Joaquin Valley Consortium of Health Officers (SJVHO), and Southern California Health Officers (SCHO) (Figure 2) (3). An initial recommendation of the COVIDNet Expert Panel Advisory Group was to target 2 to 5% of the SARS-CoV-2 positive samples in California for sequencing, provided that sampling was random and that less heavily populated counties, such as those in the RANCHO region, were well-represented. To gauge representativeness across the state, we compared sequencing rates from each Health Officers region on a per 100,000-person basis.

### 2.5. California COVIDNet laboratory network

Success of COVIDNet required establishing a network of diagnostic and sequencing laboratories with varied and critical roles. CDPH developed a Memorandum of Understanding form entitled the “COVIDNet Laboratory Participation Agreement,” whereby participating laboratories became official COVIDNet partners to serve as diagnostic, processing (for viral nucleic acid extraction), or sequencing laboratories. Public, private, and academic laboratories became part of this network.

### 2.6. Scaling sequencing capacity and sample storage

Early in the pandemic, CZB provided free COVID-19 testing and SARS-CoV-2 WGS services, technical consultation, and bioinformatic resources to CDPH, LHJs, and local PHLs. The WGS data generated from CZB served as decisive proof-of-concept that a comprehensive genomic surveillance program for SARS-CoV-2 in California could support and inform public health action and policy. Soon thereafter, the University of California Office of the President (UCOP) and CDPH partnered to quickly establish contracts with eleven UC laboratories to provide WGS capacity services to scale sequencing to at least 5,000 genomes per week. Adding to this capacity were several private laboratories also contracted to provide sequencing services. CDPH established an onsite high-throughput workflow to receive and process thousands of SARS-CoV-2 positive samples every week, extracting and transporting SARS-CoV-2 viral RNA to COVIDNet laboratory partners for sequencing. The standardized extraction protocol, in addition to the standardized analytic pipeline (described below), supported quality control comparisons among the different sequencing methods used by the contracted WGS laboratories.

Aliquots of SARS-CoV-2 samples sent out for sequencing were archived for long-term storage at-80°C. We had to purchase ten additional-80°C freezers to accommodate storage capacity needs due to the large influx of samples during Delta and Omicron surges.

### 2.7. Centralized repository for sequencing data and designating variants

As previously described (4), CDPH established a centralized sequence repository (COVIDNet sequence database) and analysis structure for SARS-CoV-2 data using cloud storage and computation capabilities (Terra.bio on the GCP) in line with recommendations outlined by Black et al. (5). To ensure CDPH access to SARS-CoV-2 sequence data generated from samples within the state but sequenced by non-COVIDNet partners, the California Code of Regulations (CCR) Title 17 Section 2505 was updated in July 2021 to require that SARS-CoV-2 lineage/variant results including Global Initiative on Sharing All Influenza Data or GISAID (6–8) reference number or raw sequence data, from any California-sourced positive specimen, be reported to CDPH by the sequencing laboratory^2^ (9). Sequencing laboratories were provided with an SFTP route to upload FASTQ files and metadata securely to the COVIDNet sequence database and separate metadata repository, respectively. The majority of non-COVIDNet sequence data was submitted to the COVIDNet sequence database by laboratories under contract with the U.S. Centers for Disease Control and Prevention (CDC). Sequence data in the COVIDNet sequence database were processed using a standardized workflow established by Theiagen Genomics, as described by Smith et al. (4). After processing, assembled genomes were uploaded to the public repositories, GISAID or National Center for Biotechnology Information (NCBI) (10).

### 2.8. Development of analytic tools to support epidemiologic analysis

The UCSC Pathogen Genomics Center was contracted to develop specialized genomic data analytic tools such as on-demand comprehensive phylogenetic resources for CDPH and LHJs, allowing rapid identification and tracking of variants and mutations of interest. UCSC deployed its Ultrafast Sample placement on Existing tRee (UShER) framework (11) to add, in near real-time, every newly sequenced sample from the COVIDNet sequence database to a global phylogenetic tree representing all available genome sequence data from public repositories (currently more than 16 million SARS-CoV-2 genomes). The global phylogenetic tree serves as the source of sequence data to build the California Big Tree – a collection of all California-sourced SARS-CoV-2 sequence data represented in a phylogenetic format^3^ (12). UCSC also developed Cluster Tracker, a geo-genomic visualization tool to predict origins of identified genetic clusters^4^ (13). This is an exploratory tool that enables users to identify introductions of SARS-CoV-2 into California (state-level, or for authorized users, county-level) and track the geographic clustering of specific SARS-CoV-2 variants or lineages. The tool displays geographic region, sample count within the cluster, clade, lineage, specimen identifiers, and timeframe of clusters based on date of specimen collection. The tool calculates metrics such as a growth score, and best potential origins and indices. Significantly, the user can click on a link to access another UCSC tool called Big Tree Investigator^5^ (14), which uses NextStrain (15) to build a phylogenetic tree around a selected cluster and enables linking of California sequences to comprehensive patient-level data reported through the California Reportable Disease Information Exchange (CalREDIE) Electronic Laboratory Reporting (ELR) and non-ELR surveillance systems. These data, displayed together, will enable the authorized CDPH or LHD users to further investigate COVID-19 transmission dynamics within the state and in some cases beyond the state. We expect Big Tree Investigator to go live in December 2023.

### 2.9. Acquisition, processing, and sequencing of samples

#### 2.9.1. SARS-CoV-2 positive specimens

In general, positive samples detected by molecular methods, including real-time reverse transcription polymerase chain reaction (RT-qPCR), loop-mediated isothermal amplification (LAMP), and transcription-mediated amplification (TMA) assays, were accepted for sequencing. Suitable maximum cycle threshold (Ct) values ranged from 28 to 33. Although Relative Light Unit (RLU) values from transcription mediated amplification (TMA) tests do not correlate directly with viral RNA concentration, TMA specimens were deemed acceptable for WGS if RLU >1,100. Later in the pandemic, as molecular testing rates declined, and antigen testing became more common, reactive swabs from antigen tests were also accepted for sequencing. COVIDNet sequencing prioritized at-risk and vulnerable populations, (e.g., congregate settings such as skilled nursing facilities, prisons, and schools) and known outbreaks, as well as striving to meet equitable representativeness across the state. COVIDNet local PHL partners contributed to these overall goals and many of them prioritized jurisdictional-based investigations of suspected re-infection and vaccine-breakthrough cases and possible importation of new variants from international travelers.

Due to high testing volume and space limitations, many diagnostic laboratories initially discarded SARS-CoV-2 positive specimens before they could be captured for sequencing. To remedy this, in April 2021, CDPH, with the support of LHJs, modified the CCR Title 17 Section 2505 to require diagnostic laboratories to provide COVID-positive remnant specimens to CDPH or a local PHL upon written request^6^ (16). Additionally, a centralized COVID-19 testing laboratory, established by the State of California, provided a pipeline of representative COVID-positive specimens collected from more than 7,000 community-based CA-TTF testing sites for WGS. This laboratory aimed to sequence all COVID-positive samples with Ct values less than 33 but ceased operations in May 2022. Additional specimen sources for WGS included those tested by local PHLs that were either sequenced onsite or by a COVIDNet sequencing partner laboratory.

To maintain compliance with California regulations related to personally identifiable information (PII) and protected health information (PHI), samples were de-identified and assigned a 9-or 10-digit Patient Anonymized Unique Identifier (PAUI) to serve as sample identification for sequence data to be processed in the Terra.bio cloud-based platform, uploaded to public repositories such as GISAID and NCBI, and subsequently linked with epidemiologic information in a secure PII/PHI-compliant environment. The PAUI numbers were coded such that the first digit corresponded to particular sample sources or projects in order to distinguish community surveillance samples from samples collected for high priority sequencing or outbreak investigations.

#### 2.9.2. Sample processing and whole genome sequencing

Samples received by CDPH (in either viral transport medium or molecular transport medium) were processed using the KingFisher Flex Purification System (Thermo Fisher Scientific, Waltham, MA United States) nucleic acid extraction platform. Briefly, the lysis step was performed within a class II biological safety cabinet by adding 275 μL of lysis solution containing binding solution and magnetic beads to 200 μL of sample in a 96-well deep well plate. After 10 min of lysis/binding, the plate was loaded onto the KingFisher® instrument along with wash plates, tip comb, and elution plate. Extracted nucleic acid was eluted in 50 μL of elution buffer. Extracts were stored at-70°C and shipped on dry ice to COVIDNet sequencing partners on a weekly basis. Samples that were tested by the LAMP method (Color Health, Burlingame, CA United States) were extracted at the testing facility as follows: total RNA was extracted using the Chemagic® 360 automated system (Perkin-Elmer, Waltham, MA United States). Samples were resuspended in 950 mL lysis buffer (CMG-832). 300uL of lysate was mixed with 300uL of 1x PBS and 10uL of polyA (CMG-842/CMG-843) and 150 uL of magnetic beads (CMG-7000), extracted per manufacturer’s protocol, eluted in 80uL of Nuclease-Free Water^7^ (17), and shipped on dry ice to CDPH as nucleic acid extracts. The State’s COVID-19 testing laboratory performed nucleic acid extractions using the Chemagic® extraction platform as described above^8^ (18). COVIDNet partner PHLs that performed their own sequencing followed nucleic acid extraction protocols compatible with their sequencing protocols.

Because of the varying capabilities and instrumentation available among the COVIDNet sequencing laboratory partners, library preparation and sequencing protocols used were dependent upon the particular sequencing method employed by the laboratory (Table 1). Sequencing library preparation methods included standard ARTIC v.3, v.4, and v.4.1 (19, 20), ARTIC v.3 with 275 bp tailed amplicons^9^ (21), the SWIFT protocol (23), Midnight protocol (25), and Varskip (24). Sequencing technologies included Illumina (San Diego, CA United States) MiSeq®, Illumina NextSeq®, Illumina NovaSeq®, AVITI™ (Element Biosciences, San Diego, CA United States), and Clear Dx (San Carlos, CA United States) (22). Illumina sequencing included single-end as well as paired-end protocols.

**Table 1:** SARS-CoV-2 sequencing protocols employed by California COVIDNet WGS laboratory partners.

Sequencing protocol	Number of COVIDNet laboratories
ARTIC or modified ARTIC^a^	27
Clear Dx^b^	11
Swift^c^	3
Varskip^d^	2

a ARTIC v.3, v.4, and v.4.1 (19, 20), ARTIC v.3 with 275 bp tailed amplicons (21).

b Clear Dx (22).

c SWIFT protocol (23).

d Varskip (24).

### 2.10. Data management: processing, analysis, and storage

Since it was not feasible to standardize the library preparation and sequencing methods by the various COVIDNet contributors, homogeneity of analysis was achieved by having all sequence data centralized and analyzed using a standardized workflow. Uniform workflow, bioinformatics analytics, and training resources were established (4) utilizing Terra.bio^10^ (26), as the centralized location to house COVIDNet sequence data.

Raw sequence data reads, in FASTQ file format, were made available through various methods on Terra.bio. County PHLs shared FASTQ files from other cloud-based platforms such as Illumina’s BaseSpace (San Diego, CA) and the Clear Labs Portal (San Carlos, CA). Read data hosted on Illumina’s BaseSpace platform were made available on Terra.bio through the BaseSpace_Fetch workflow^11^ (27), while reads stored on the Clear Labs Portal were made available on Terra.bio directly through the portal’s user interface. Academic COVIDNet partners were provisioned with GCP buckets to serve as persistent storage. They uploaded FASTQ files directly to these buckets, and the data stored in these GCP buckets were made accessible on Terra.bio through an automated cron job which ran once daily. Alternatively, FASTQ files were manually uploaded from a local machine to Terra.bio.

Once the FASTQ files were available on Terra.bio, genome assembly and characterization were performed using the TheiaCov Workflows for Genomic Characterization. This open-source workflow series is available on the Public Health Viral Genomics Github repository^12^ (28) and includes workflows for analysis of Illumina paired-end, Illumina single-end, Oxford Nanopore (Oxfordshire, England), Element Biosciences AVITI (San Diego, CA), and Clear Labs SARS-CoV-2 data. In addition to assembling the genome, these workflows also provided quality control metrics, Pango lineages (29), and Nextclade clades (15). The StaPH-B docker image for Pangolin^13^ (30, 31) was used within the TheiaCov workflow, wherein the UShER (11) mode of Pangolin was used by default for lineage assignment. Whenever the docker image was updated following a pangolin-data^14^ (32) release, the Pangolin_Update workflow^15^ (33) on Terra.bio was run to assign updated lineages to California sequences with collection dates in the past two months.

SARS-CoV-2 sequence data from CDPH were submitted to GISAID and NCBI if the genome assembly covered at least 83% of the Wuhan-1 reference genome (MN908947) (34), as determined by the TheiaCov workflows. The Mercury workflow series on Terra.bio, also hosted within the Public Health Viral Genomics Github repository (28) were used to reformat the FASTA files and metadata according to the submission guidelines for GISAID and NCBI. Genome assemblies from CDPH were uploaded to GISAID and GenBank (35) and raw reads were uploaded to the Sequence Read Archive (SRA) (36) with the exception of data generated on the Element Biosciences AVITI instrument, as data from that instrument cannot be accepted at this time. Raw reads uploaded to CDPH SARS-CoV-2 BioProject (PRJNA750736) were depleted of host reads using the SRA human read scrubber^16^ (37). All California local PHLs that used Terra.bio also uploaded to GISAID and NCBI using their own quality control thresholds for submission. It is important to note that samples sequenced by local PHLs were distinct from the samples sequenced by COVIDNet sequencing laboratories and therefore duplicate submissions to public repositories were highly unlikely and not considered a concern. The Terra.bio platform also allowed local PHLs to customize and optimize workflows for their own use, such as to build automated import and export pipelines to decrease reliance on manual processes.

Data on Terra.bio are exported to external Google buckets for downstream visualization, alerting, and reporting using the Terra_2_BQ workflow^17^ (38). Data were then ingested into Big Query projects using either a cron job running a shell script, or a Google workflow. An SQL query was used to combine data from all COVIDNet partners into a single data source. Looker, a GCP for automated alerts and reports, or Looker Studio, a visualization platform, was used to monitor the changes in SARS-CoV-2 lineages over time.

### 2.11. Quality of sequence data

Successful sequencing was determined by “percent reference coverage,” i.e., the proportion of the genome successfully sequenced to a minimum depth of 20X. The minimum percent reference coverage required for uploading to a public sequence repository was 83%, approximately 25,000 bases of a SARS-CoV-2 genome. Other quality metrics assessed, but not necessarily used to reject sequences, included: (1) the quantiles of sequencing depth across the results of a sequencing run; (2) the proportion of putative human DNA (as found via Kraken2 (39) analysis) vs. the percent reference coverage; and, (3) sample or within-run contamination, as evidenced either by high proportions of apparent minor allele frequency single nucleotide polymorphisms (SNPs) across samples in a sequencing run or by improbable phylogenetic placement, such as detection of a variant from samples collected prior to the emergence of said variant. More recently (as of April 2023), samples with a disproportionately high number of variants (>17.5%) with allele frequencies between 60 and 90% were rejected due to the likelihood that such samples were either contaminated or coinfected. We assumed that contamination was more likely in these cases than coinfection, and that coinfections, if present, would be rare and unlikely to affect the understanding of surveillance significantly. The lower threshold of 60% was selected because it is also the minimum threshold for calling a variant allele for assembly and thus may have affected lineage determination and phylogenetic placement.

## 3. Results

One primary goal of COVIDNet was to sequence 2–5% of SARS-CoV-2 positive samples in California, in a representative and equitable manner. Through collaborative agreements and contracts with a variety of partners, we successfully engaged 50 laboratories including public health, academic, and private laboratories to achieve large-scale WGS of SARS-CoV-2 throughout California. As of March 2023, CDPH has received more than 660,000 samples from various submitters throughout the state and processed and extracted more than 217,000 samples (data not shown) that met WGS criteria (e.g., Ct value <33 or from reactive antigen tests). As the pandemic progressed and the COVIDNet workflow became routine, we expanded SARS-CoV-2 genomic surveillance to include (1) testing sites along the international border between California and Mexico, (2) at three international airports, (3) at community organizations serving priority populations, and (4) at all schools participating in the CA-TTF testing program. In mid-June 2021, we partnered with a large integrated health system that serves over 4.5 million members in Northern and Central California (40) to characterize SARS-CoV-2 variants in both inpatient and outpatient populations, further expanding the COVIDNet surveillance network. In the latter part of 2022, as antigen testing began to predominate over molecular testing for SARS-CoV-2, select COVIDNet partners transitioned WGS operations to accept swabs from reactive antigen tests to maintain an adequate level of surveillance as much as possible.

Through COVIDNet, WGS capacity and bioinformatics capability increased within the network of 29 California PHLs at the state and local levels. By the end of 2021, a total of 15 (52%) of 29 PHLs were conducting SARS-CoV-2 genomic surveillance via COVIDNet. To date, WGS capacity for SARS-CoV-2 has been established at 19 of the 29 (66%) PHLs in the state. Six California PHLs have hired bioinformaticians for SARS-CoV-2 sequence analysis. In mid-2022, CDPH formally established a new Genomic Epidemiology Section to analyze, manage, and apply SARS-CoV-2 genomic surveillance data for situational awareness, and to inform infectious disease modeling and forecasting^18^ (41), public health action, and policy.

### 3.1. Sequencing volume

**Figure 3:**
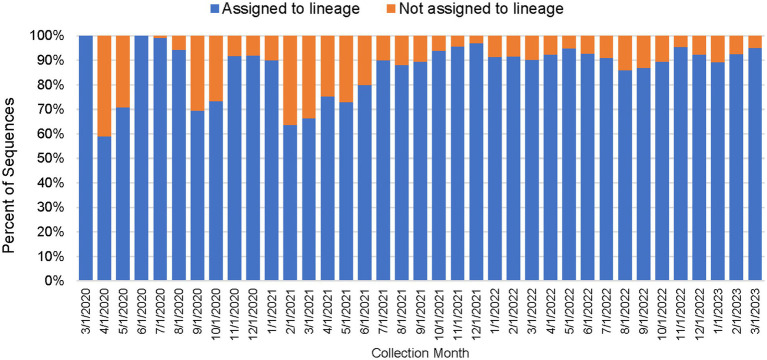
Percentage of SARS-CoV-2 sequences in the California COVIDNet sequence database assigned to a lineage or not assigned (March 2020 to March 2023).

**Figure 4:**
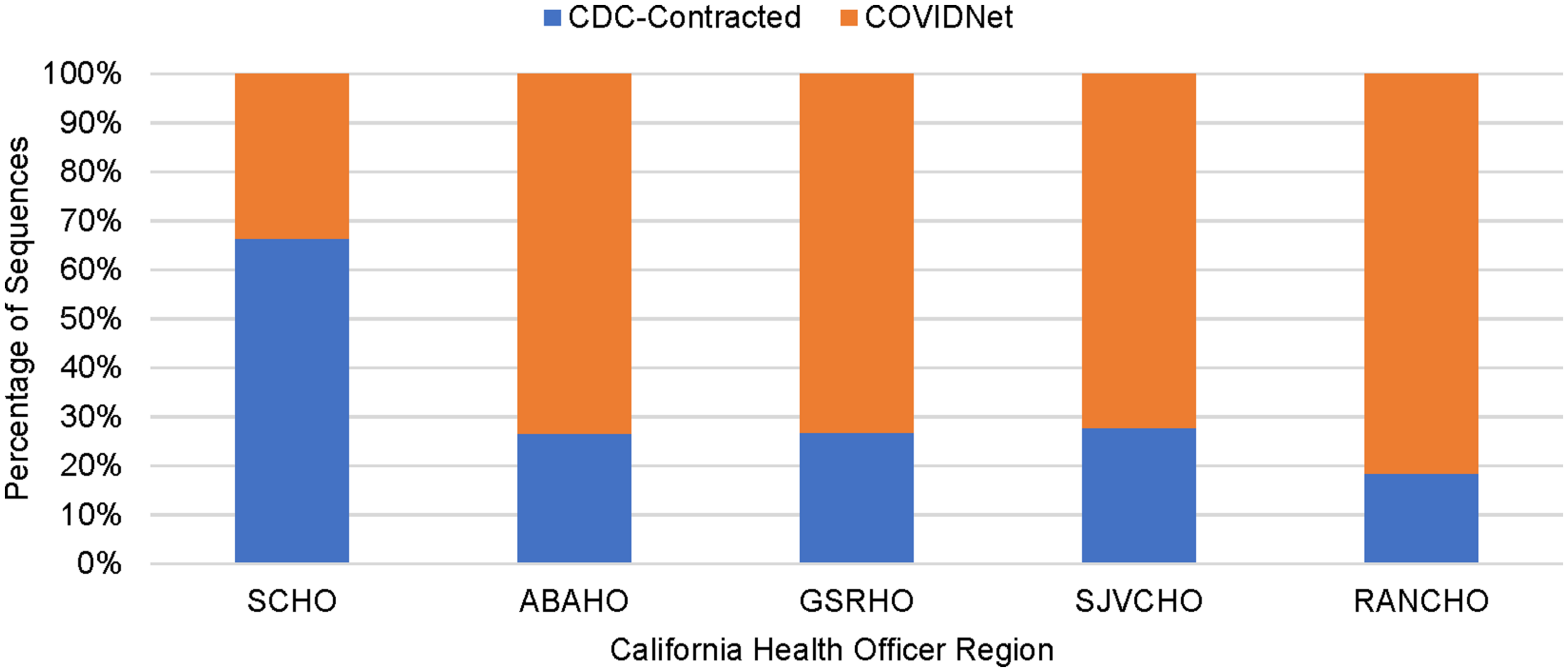
Percentage of sequenced samples for each California Health Officers Region by laboratory category (March 2020 to March 2023).

As shown in Table 2, between March 2020 through March 2023, a total of 450,030 genomes were deposited by COVIDNet partner laboratories into the California COVIDNet sequence database in Terra.bio, with 344,837 genomes (77%) meeting the 83% reference coverage threshold to upload to GISAID or NCBI. The percent of genomes uploaded was higher overall for PHLs (87%) than for COVIDNet contract laboratories (74%) (Table 2). In some cases, this was likely due to differences in specimen handling. Samples processed at the COVIDNet contract laboratories typically underwent several freeze-thaw cycles prior to extraction, had to be transported to CDPH for extraction, or were of poor quality. The percentage of sequences assigned to a lineage increased over time and has remained above 80% since July 2021 (Figure 3). From March 2020 through March 2023, CDC-contracted laboratories contributed more than 335,000 sequences to the California COVIDNet sequence database (data not shown). Most of the California-sourced samples (~75%) sequenced by CDC-contracted laboratories were from the Southern California Health Officer (SCHO) region (data not shown), the most populous region of the state (Table 3). Samples sequenced by CDC-contracted laboratories made up 66% of samples from the SCHO region in the COVIDNet sequence database, compared to less than 30% from the four other regions (Figure 4). In regions other than the SCHO, more than 50% of the samples were sequenced by COVIDNet laboratory partners (Figure 4).

**Table 2:** SARS-CoV-2 genomes stored in the California COVIDNet sequence database (March 2020 through March 2023).

Laboratory Category	Total genomes sequenced	Total genomes uploaded^*^ and accepted^#^	Percent of genomes uploaded^*^ and accepted ^#^
COVIDNet contract	356,875	263,706	74%
Public Health Laboratory (State and local)	93,155	81,131	87%
Totals	450,030	344,837	77%

*Genomes that achieved ≥ 83% reference coverage were uploaded to GISAID or NCBI sequence repositories. #A genome was considered accepted by GISAID if it was released upon curation without requiring further confirmation or modification of the sequence data.

**Table 3:** SARS-CoV-2 sequencing volume by California Health Officers Region and population (March 2020 – March 2023).

California (CA)Health Officersregion	Population (2021)	Percent of CA population	Number of sequences	Percent of sequences	Number of sequences (per 100,000 population)
ABAHO	8,451,422	21%	163,948	24%	1,940
GSRHO	2,964,755	8%	51,489	7%	1,737
RANCHO	701,548	2%	14,631	2%	2,086
SJVCHO	4,470,528	11%	86,492	13%	1,935
SCHO	22,867,100	58%	377,387	54%	1,650
California Total	39,455,353	100%	693,947	100%	1,783
ABAHO: Association of Bay Area Health Officials					
GSRHO: Greater Sacramento Region of Health Officers					
RANCHO: Rural Association of Northern California Health Officers					
SJVHO: San Joaquin Valley Consortium of Health Officers					
SCHO: Southern California Health Officers					

**Figure 5:**
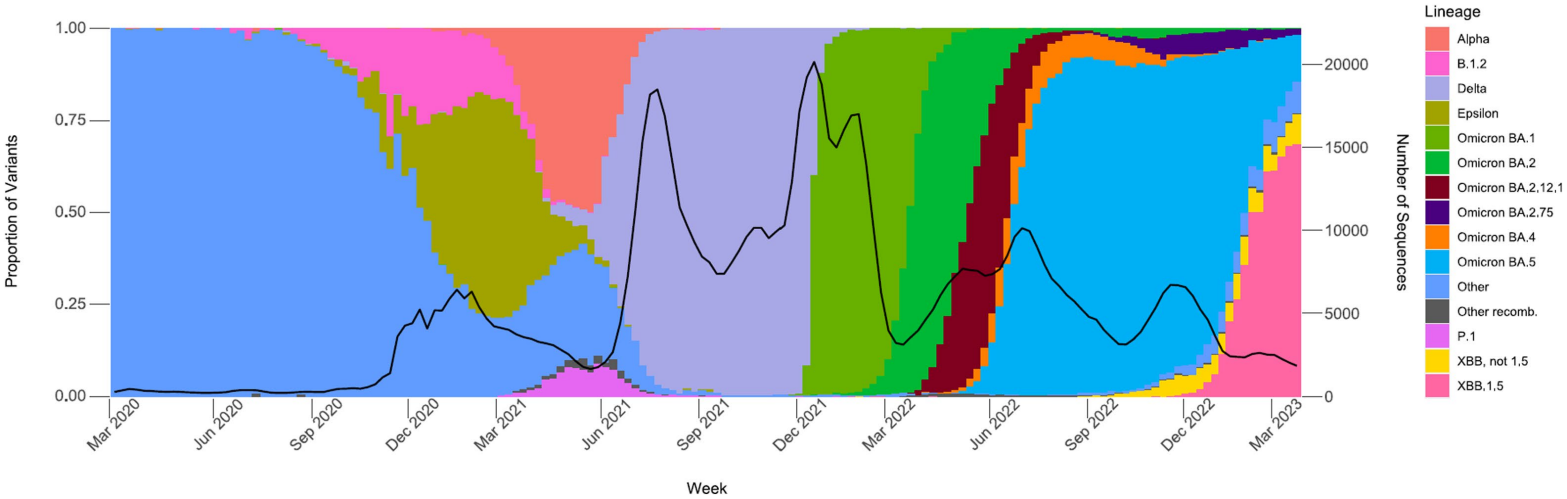
Proportions of major SARS-CoV-2 variants by collection date from the California COVIDNet sequence database, March 2020 through March 2023. Data graphed represent weekly proportions with the black line representing the 4-week rolling average number of samples sequenced. Colors correspond to indicated SARS-CoV-2 variant lineage.

Between March 2020 and December 2020, an average of 361 samples were sequenced per month, with an average of 0.7% of positive tests sequenced (data not shown). From January 2021 through June 2021, a time period that encompasses the start of COVIDNet sequencing in March 2021, an average of 20,875 samples were sequenced per month, with an average of 7.9% (~11-fold increase from 2020) of positives sequenced. The large-scale genomic surveillance efforts across California by COVIDNet and CDC-contracted laboratories has resulted in nearly 800,000 SARS-CoV-2 genomes in the COVIDNet sequence database. Between March 2020 and March 2023, these data have allowed us to monitor the emergence and spread of different variants statewide including the emergence of Epsilon in the fall and winter of 2020, followed by co-circulation of Alpha and P.1 (Gamma) in the spring of 2021 (Figure 5). Later we observed the transition to Delta in June 2021 followed by the abrupt introduction and predominance of Omicron BA.1 in December 2021 with subsequent diversification of Omicron sublineages, and dominance of BA.5 from June 2022 into January 2023. We saw the rise of the XBB and other recombinants in late winter of 2022 with XBB.1.5 predominating at the end of March 2023 (Figure 5).

**Figure 6:**
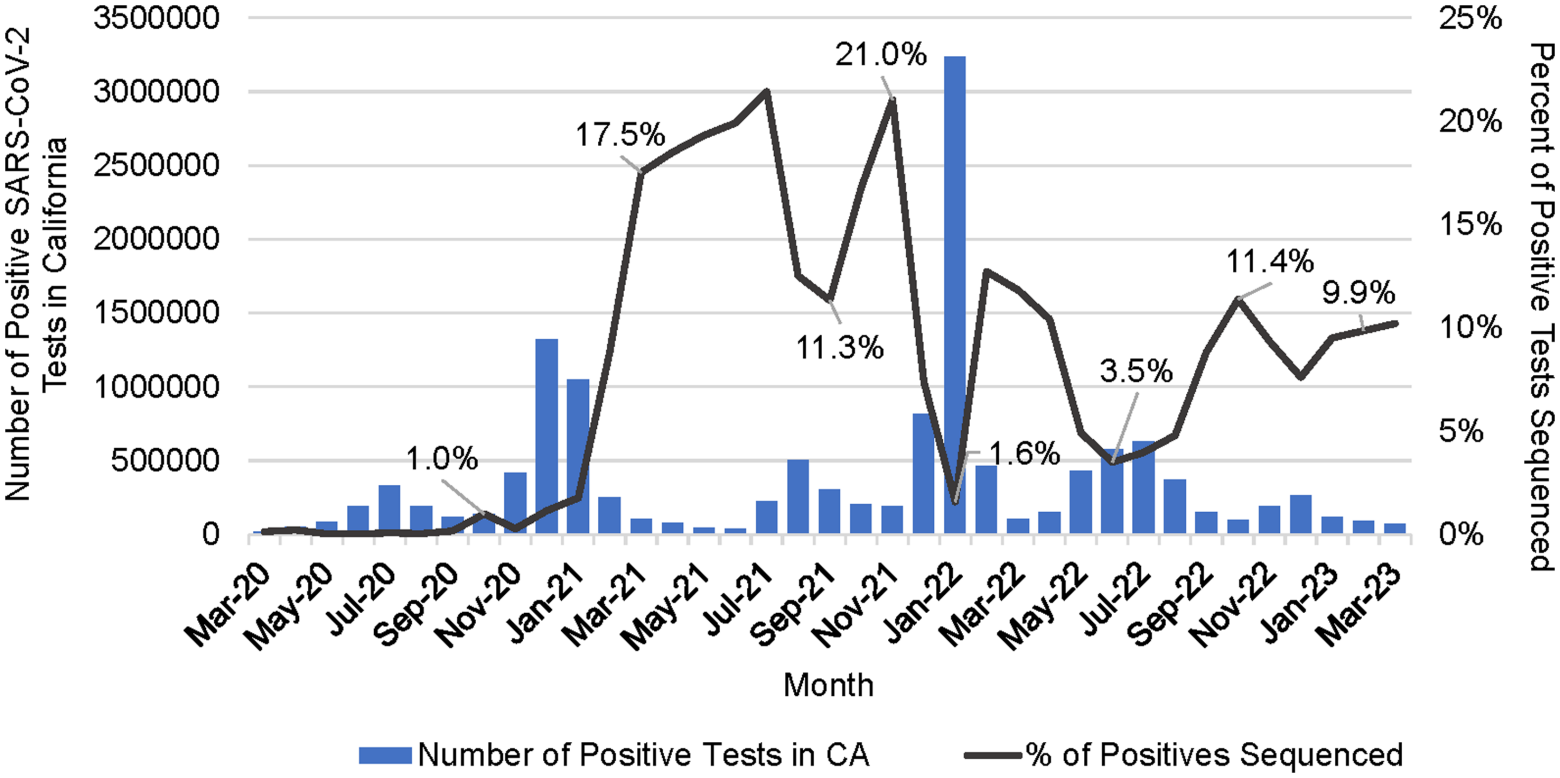
Number of positive SARS-CoV-2 tests in California (CA) per month and the percent of positive tests that were sequenced per month (March 2020 to March 2023).

The percentage of positive samples sequenced peaked at over 21% in July 2021 (Figure 6). Sequencing volume peaked between July 2021 and February 2022, averaging 52,941 samples per month, which correlated with a spike in the number of reported positive tests in the state. The percent of positives sequenced averaged 7.1% but dropped to a low of 1.6% in January 2022 during the Omicron surge (Figure 6). From March 2022 through January 2023, sequencing volume declined to an average of 16,881 sequences per month, and an average of 6% of positive tests sequenced.

### 3.2. Sequencing representativeness

**Figure 7:**
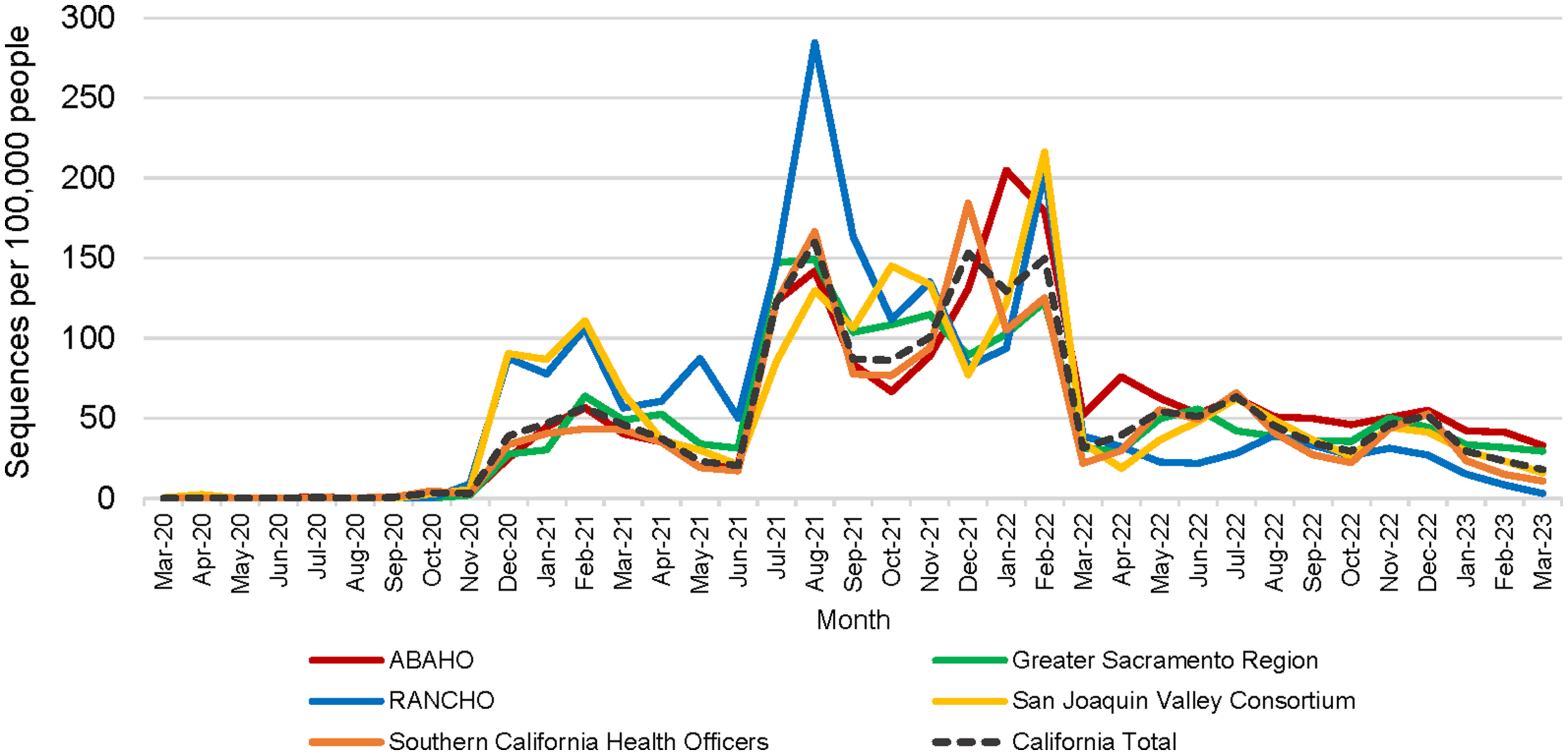
Number of SARS-CoV-2 sequences per 100,000 people by month for each California Health Officers region (March 2020 to March 2023).

All 61 California health jurisdictions were represented within the COVIDNet sequence database. Sequencing representativeness was similar across the five Health Officers regions of California. The number of samples sequenced per 100,000 people ranged from 1,650 to 2,086 from March 2020 through March 2023 (Table 3). This value varied over time, but in total remained similar across Health Officers regions at specific time points, except for a spike in August 2021 from the RANCHO region (Figure 7). The proportion of sequences per respective Health Officers region corresponded approximately well with each region’s population percentage (Table 3). Given that nearly two-thirds of the sequences generated by CDC-contracted laboratories were from the SCHO Region, sample representativeness across other regions in the state was assured by supplementing with COVIDNet sequencing of samples from CA-TTF community-based sites and other sources (Figure 4).

### 3.3. Quality of sequence data

We routinely monitored the quality of sequence data generated by the COVIDNet sequencing laboratories which varied across the network (data not shown). We met with the sequencing laboratories monthly to review overall sequencing quality and success of individual sequencing runs; occasionally we requested re-sequencing when a high proportion of failures or evidence of significant contamination was detected. Although the measure of human DNA vs. the percent reference coverage was not used as a criterion for rejection, low percent reference coverage (i.e., failed sequencing) tended to strongly correlate with a high proportion of human DNA in a sample. While the overall sequencing quality was acceptable across COVIDNet partners (as represented by proportion of sequences assigned a lineage designation) (Figure 3), some laboratories experienced sporadic issues requiring re-sequencing and root-cause analysis to prevent recurrent quality excursions (data not shown). Problems included general quality issues such as unacceptably low numbers of samples passing quality metrics on a given sequencing run, as well as occasional detection of contamination from a sample or within a sequencing run, as evidenced either by high proportions of minor allele frequency SNPs across samples within a sequencing run, or for example, by the improbable detection of putative Omicron sequences from samples collected prior to the known date of Omicron emergence. In some instances, we observed declines in sequence quality over time as the virus evolved which indicated the need to update sequencing protocols or primers, particularly with the emergence of the Omicron variant in November 2021. In general, the goal of data quality monitoring was to keep the number of errors in sequences released to public repositories low, while not slowing throughput needed to maintain relevant and current surveillance.

## 4. Discussion

### 4.1. Public health impact of COVIDNet

The large-scale approach to genomic surveillance implemented by California COVIDNet partners and others has enabled CDPH and LHDs to monitor the evolution of SARS-CoV-2 over time including transitions between viral variants of interest and variants of concern (VOC). COVIDNet efforts have provided valuable data supporting epidemiologic investigations and policy-making decisions in California, at the state and local levels. COVIDNet data have revealed local trends of viral transmission, helped to characterize and better understand outbreaks of SARS-CoV-2 within skilled nursing facilities, schools, and other settings (42–50) and have provided situational awareness of circulating variants with the potential to impact efficacy of vaccines and therapeutics. Of particular note was the use of WGS data to assess the levels of circulating SARS-CoV-2 variants with known resistance to monoclonal antibodies that effectively ruled out such treatment. Furthermore, COVIDNet data have enabled identification and characterization of cases and variants associated with vaccine breakthrough infections, the first cases of new VOCs in the state, re-infections (40) (45) (49), and long-term infections demonstrating intra-host evolution (unpublished). Regardless of publication status, sequence data meeting quality criteria have been uploaded in a timely manner to data repositories for public access. COVIDNet efforts have provided data enabling California to establish its own COVID forecasting model available online to the public^19^ (41).

The cloud-based data infrastructure developed to store the large volume of COVIDNet WGS data and to provide a framework for analysis has created capability, not only for SARS-CoV-2 but also other pathogens, such as monkeypox virus (MPXV), enteric bacteria, and select pathogens associated with hospital infections. The workflows for SARS-CoV-2 sequence analysis have been leveraged for wastewater surveillance (WWS) applications and will be utilized in the continued expansion of WWS at CDPH. Using automated tools (e.g., Google Looker) to query the COVIDNet sequence database at defined time periods, we set up email alerts to notify relevant public health officials about the detection or emergence of concerning variants, mutations of interest, and proportions of variants detected weekly (4).

In April 2022, as the COVID-19 pandemic continued, the emergence and subsequent global spread of MPXV occurred (51–53). Although WGS protocols and data analysis for this emerging pathogen were not in place at that time, we were able to modify the SARS-CoV-2 WGS workflow and adapt the COVIDNet data analysis infrastructure for timely MPXV sequence analysis. MPXV WGS results were shared among California LHDs and PHLs to examine transmission patterns and evolution of MPXV as it spread in different regions of the state.

### 4.2. Accomplishments

COVIDNet successfully achieved many of its original goals and objectives. We achieved large-scale genomic surveillance of SARS-CoV-2 across California, which allowed us to monitor the emergence and spread of variants statewide including the lineage diversification of Omicron variants and the rise of SARS-CoV-2 recombinants (Figure 5). Nineteen of 29 California PHLs have established WGS capability for SARS-CoV-2 and are now thusly prepared for future public health crises and pandemics. Due to the efforts of both COVIDNet partners and non-COVIDNet laboratories, there are nearly 800,000 genomes in the COVIDNet sequence database, and as cases of COVID-19 continue to occur, this number will continue to increase.

With COVIDNet, we established significant collaborations with academic and private partners that strengthened statewide capacity to respond to COVID and future infectious disease threats. We will endeavor to maintain these important partnerships to benefit and ensure preparedness for public health. The success of COVIDNet demonstrates the power of productive collaborations among California’s public, private, and academic institutions in responding to an unprecedented international public health emergency. The response to COVID-19 in California laid the foundation for COVIDNet, as a system, to be adapted for other pathogens of public health importance and future public health emergencies.

### 4.3. Challenges

The accomplishments of implementing COVIDNet did not occur without challenges, some resolved, some still ongoing, with many inherent to outdated public health infrastructure and data systems. Challenges included navigating hierarchies of data management needs from samples, sequences, metadata, and results. Because COVIDNet receives disparate data from multiple sources and projects, we were forced to develop multiple databases to manage incoming data and samples amid viral pandemic surges. This approach allowed us to quickly scale during the pandemic, but it created burdensome processes for managing and analyzing data from the variety of sources. It also caused inconsistencies and redundancies in data, such as duplicate sequences with different internal identifiers, inability to easily manage multiple samples from single individuals, and incomplete metadata.

Patient-level metadata is frequently classified PII and PHI that cannot be housed in the same location as its matching viral genomic data. The COVIDNet sequence database is specifically a PII/PHI-free cloud-based platform, consistent with this tenet. It is a significant challenge to join sequence data with PII/PHI data due to incompatibilities between the two data systems and associated privacy regulations. Substantial investments were made in collaboration with UCSC to develop a tool, Big Tree Investigator, to automate integration of sequence data from the COVIDNet sequence database with patient-level data located in a separate secure PHI-compliant environment. Big Tree Investigator advances beyond earlier genomic epidemiology tools to facilitate linking of these databases to visualize sequence data with associated PHI-metadata mapped on a phylogenetic tree. Such visualizations will provide context from other sequences and metadata to understand clusters and outbreaks to, possibly, contain/control further transmission within a defined community or region (i.e., conduct genomic epidemiology). Developing this tool has been impeded by complications relating to PII/PHI concerns and limited assets, but we anticipate that Big Tree Investigator will go live in December 2023.

### 4.4. Limitations

The COVIDNet project had several limitations, many of which still exist. First and foremost was the static and risk-averse nature of public health infrastructure and systems at the state and local levels that were insufficient to support advances in sequencing technology and attendant data requirements, particularly during the chaos of the pandemic. Prior to the pandemic, funding for public health had declined and was usually insufficient to implement modern systems or needed updates. The pandemic resulted in increased funding for public health response, but in many cases the infrastructure was a hindrance in putting the funds to use quickly or optimally.

A major limitation was achieving timely generation of sequence data. Contracts with COVIDNet partners established a 2-week turnaround time for sequence results but delays of 2 weeks or more to process and ship samples out to COVIDNet partners were typical, impeding timely results. Although CDPH can provide rapid SARS-CoV-2 WGS (49), this was not scalable and proved useful only for certain situations, such as high-risk outbreaks or severe cases.

Limitations in sampling strategies to meet goals of representativeness and equity were manageable because of widespread availability and accessibility of COVID-19 testing from established community-based testing sites and other sources. The closure of these testing sites, as well as the transition from molecular-based to antigen-based testing made it difficult to maintain the goal of geographic and equitable representation and we expect this challenge to continue. As sources of samples for sequencing decrease, the risk of surveillance bias will also increase. Given the success of SARS-CoV-2 wastewater surveillance (54–56), we expect wastewater surveillance to continue to contribute a significant portion of data to inform SARS-CoV-2 genomic surveillance going forward and that this will help to mitigate surveillance bias.

A further limitation is that, although we established a PAUI system to distinguish between surveillance samples and other high priority/outbreak samples, the COVIDNet database contains sequences that do not have PAUI numbers and thus it is not always clear whether these samples are surveillance-based or from targeted investigations and therefore not randomly selected. Thus, outbreak specimens and specimens from high-risk settings may be over-represented as surveillance data. Likewise, the vast majority of specimens that were sequenced did not include attendant detailed medical or travel history, and thus this genomic surveillance program was primarily laboratory-based.

### 4.5. Recommendations and conclusions

As we move to the next phase of COVID-19, it is important to ensure funding to support ongoing genomic surveillance for SARS-CoV-2 and other pathogens of public health significance globally, nationally, and at state and local levels. Secure and sustained funding is critical to ensure the capacity to identify and analyze emerging pathogens of concern. Resources are also required to address gaps in public health infrastructure, in particular, systems related to data management, storage, and analysis. These systems are in significant need of modernization and functional interoperability to optimize data transmissions.

To build on experience gained with COVIDNet and prepare for future crises, it is imperative that we create, in advance, uniform data requirements with the ability to integrate incoming data from many sources, and transition to database and query platforms that can handle very large datasets.

Partnerships between public health departments and clinical laboratories should be established to help enhance collaborations and prepare us for the next large outbreak or pandemic. By enhancing these partnerships, we can help to identify and submit specimens for genomic surveillance as part of routine public health surveillance systems and also provide critical genomic information for the medical community that might help guide care and treatment. Protocols should be developed between these institutions to create a sample referral system in the event of outbreaks of concern or public health emergencies so that specimens are not discarded before they can be captured for sequencing or other characterization.

The value of COVIDNet will continue with ongoing genomic surveillance of SARS-CoV-2, as well as other pathogens of public health significance. Importantly, the COVIDNet infrastructure has already demonstrated, with the emergence of MPXV, its utility to be leveraged for the next public health crisis. The flexibility of this large-scale, collaborative system provides the necessary methods, logistics, and workflows that require only minor modifications to enable effective genomic surveillance and epidemiology at local, regional, and state levels.

## Data availability statement

The datasets presented in this study can be found in online repositories. The names of the repository/repositories and accession number(s) can be found at: https://www.ncbi.nlm.nih.gov/, Bioproject number PRJNA750736.

## Group members of the COVIDNet Consortium

Summer Adams, Phacharee Arunleung, Matthew Bacinskas, Nikki Baumrind, Elizabeth F. Baylis, Cynthia Bernas, John M. Bell, Ricardo Berumen, Ellen L. Bouchard, Brandon Brown, Teal Bullick, Lyndsey Chaille, Alice Chen, Giorgio Cosentino, Yocelyn Cruz, Nick D’Angelo, Mojgan Deldari, Alex Espinosa, Ambar Espinoza, Eric M. Foote, Gautham, Shiffen Getabecha, Sabrina Gilliam, Carol A. Glaser, Madeleine Glenn, Bianca Gonzaga, Ydelita Gonzales, Melanie Greengard, Hugo Guevara, Jill K. Hacker, Kim Hansard, April Hatada, Monica Haw, Thalia Huynh, Kathleen Jacobson, Chantha Kath, Paul B. Kimsey, Katya Ledin, Deidra Lemoine, Ruth Lopez, Sharon L. Messenger, Blanca Molinar, Christina Morales, Samantha Munoz, Robert Nakamura, Nichole Osugi, Tasha Padilla, Chao-Yang Pan, Mayuri V. Panditrao, Chris Preas, Will Probert, Alexa Quintana, Maria Uribe-Fuentes, Mayra Ramirez, Clarence Reyes, Estela Saguar, Maria Salas, Ioana Seritan, Brandon Stavig, Hilary Tamnanchit, Serena Ting, Debra A. Wadford, Cindy Wong, Chelsea Wright, and Shigeo Yagi, California Department of Public Health (CDPH); Venice Servellita, Alicia Sotomayor-Gonzalez, and Charles Y. Chiu, Chiu laboratory at University of California, San Francisco; Isabel Bjork, Joshua Kapp, Anouk van den Bout, and Ellen Kephart, Colligan Clinical Diagnostic Lab, University of California, Santa Cruz; Mawadda Alnaeeli, Hau-Ling Poon, Scott Topper, Color Health; Marzieh Shafii, Sara Sowko, Stephanie Trammell, and Erik Wolfsohn, Contra Costa County PHL; Patrick Ayscue, Amy Kistler, Emily Crawford, Joseph DeRisi, and Cristina Tato, Chan Zuckerberg Biohub; Valeria Arboledaz, Eleazar Eskin, and Laila M. Sathe, Department of Computational Medicine, University of California, Los Angeles; Jacek Skarbinski, Kaiser Permanente Northern California, Division of Research; Abigail Duque, Jeffrey Schapiro, and Ivy Yeung, Kaiser Permanente Northern California, The Permanente Medical Group; Rama Ghatti and Zahra Shajani-Yi, LabCorp; Jacob M. Garrigues, Nicole Green, and Peera Hemarajata, Los Angeles County PHL; Carlos Anaya and Donna Ferguson, Monterey County PHL; Beatrix Kapuszinsky, Favian Ramirez, and Felipe Sta Agueda, Napa-Solano-Yolo-Marin-Mendocino County PHL; Megan Crumpler and Julia Wolfe, Orange County PHL; Russell B. Corbett-Detig, David Haussler, Marc Perry, and Jakob McBroome, Pathogen Genomics Center, University of California, Santa Cruz; Nhi Duong, Deborah Forester, and Anthony Gonzalez, Sacramento County PHL; Maria J. Victorio, Anna Liza M. Manlutac, Jeremy Corrigan, and Nicholas S. Rhoades, San Diego County PHL; Lina Castro and Godfred Masinde, San Francisco County PHL; Harmeet Kaur, Monica Paniagua-Alexander, San Joaquin County PHL; Katrina G. Erwin, Glen Miller, and Frances N. Sidhu, San Luis Obispo County PHL; Morris Jones, Sangita Kothari, and Christopher Ngo, San Mateo County PHL; Brandon Bonin, Daniel Castillo, and Rensen Khoshabian, Santa Clara County PHL; Kristian Andersen, Mark Zeller, SEARCH Alliance; Lisa Critchett, Carlos Gonzalez, Iryna V. Goraichuk, and Rachel Rees, Sonoma County PHL; Frank Ambrosio, Curtis J. Kapsak, Kevin G. Libuit, Michelle R. Scribner, Joel R. Sevinsky, Emily A. Smith, and Sage M. Wright, Theiagen Genomics; Vanessa B. Cadiz, Denise Lopez, and Matthew Rosman, Tulare County PHL; Bryan Bach, Stacia Wyman, UC Berkeley Innovative Genomics Institute SARS-CoV-2 Sequencing Team; Charlotte Acharya, Ryan Davis, and Richard Michelmore, UC Davis Genome Center; Melanie Oakes and Suzanne Sandmeyer, UC Irvine Campus Covid Testing Laboratory & Genomics High Throughput Facility; Kathy Borkovich, Clay H. Clark, Holly Clark, and Brandon Le, UC Riverside Institute for Integrative Genome Biology - Genomics Core Facility; Peter De Hoff, Kristen Jepsen, Rob Knight, and Louise C. Laurent, UC San Diego EXCITE Laboratory; Zack Aralis and Carolina Arias, UC Santa Barbara Arias Lab; Varuzhan Balasanyan, Mark Duhon, and Xinmin Li, UCLA Technology Center for Genomics & Bioinformatics; Eric Chow, Nicole Leung, Delsy Martinez, and Tyler T. Miyasaki, UCSF Center for Advanced Technology; Ashlee Clow, Jared Hoffman, and Thomas Rush, Ventura County PHL.

## Author contributions

DW, JD, SM, KJ, JB, and RC-D conceived of this project, and guided it to fruition. DW, JB, NB, SG, ES, SM, JH, EF, ELB, CG, KL, and KJ wrote and revised the manuscript. All members of the COVIDNet Consortium contributed to data acquisition, analysis, results interpretation, analysis, results interpretation, or supervision of the work. All authors contributed to the article and approved the submitted version.

## Footnotes

^1^ https://testing.covid19.ca.gov/

^2^ https://www.cdph.ca.gov/Programs/CID/DCDC/CDPH%20Document%20Library/LabReportableDiseases.pdf

^3^ https://genome.ucsc.edu/cgi-bin/hgPhyloPlace

^4^ https://clustertracker.gi.ucsc.edu/

^5^ https://pathogengenomics.ucsc.edu/tools

^6^ https://www.cdph.ca.gov/Programs/CID/DCDC/CDPH%20Document%20Library/LabReportableDiseases.pdf

^7^ https://www.fda.gov/media/138249/download

^8^ https://www.fda.gov/media/147547/download

^9^ https://www.protocols.io/view/ucsf-cat-covid-19-tailed-275bp-v3-artic-protocol-v-kxygxpnpzl8j/v1

^10^ https://terra.bio

^11^ https://github.com/theiagen/terra_utilities/blob/main/workflows/wf_basespace_fetch.wdl

^12^ https://github.com/theiagen/public_health_viral_genomics

^13^ https://hub.docker.com/r/staphb/pangolin/

^14^ https://github.com/cov-lineages/pangolin-data

^15^ https://github.com/theiagen/public_health_viral_genomics/blob/main/workflows/wf_pangolin_update.wdl

^16^ https://github.com/ncbi/sra-human-scrubber

^17^ https://github.com/theiagen/terra_utilities/blob/main/workflows/wf_terra2bq.wdl

^18^ https://calcat.covid19.ca.gov/cacovidmodels/

^19^ https://calcat.covid19.ca.gov/cacovidmodels/
